# Low free thyroxine and normal thyroid-stimulating hormone in infants and children: possible causes and diagnostic work-up

**DOI:** 10.1007/s00431-021-03976-6

**Published:** 2021-02-13

**Authors:** Peter Lauffer, A. S. Paul van Trotsenburg, Nitash Zwaveling-Soonawala

**Affiliations:** Department of Pediatric Endocrinology, Emma Children’s Hospital, Amsterdam University Medical Center, University of Amsterdam, Meibergdreef 9, 1105 AZ Amsterdam, The Netherlands

**Keywords:** FT4, TSH, Central hypothyroidism, Children

## Abstract

Screening for hypo- or hyperthyroidism in adults is generally done by measuring the serum thyrotropin (thyroid-stimulating hormone, TSH) concentration. This is an efficient approach in case of suspected acquired thyroid disease. However, in infants and children, congenital hypothalamus-pituitary-thyroid (HPT) axis disorders also need to be considered, including primary and central congenital hypothyroidism, and even rarer thyroid hormone receptor and transporter defects. In primary congenital hypothyroidism, TSH will be elevated, but in the other congenital HPT axis disorders, TSH is usually within the normal range. Free thyroxine (FT4) assessment is essential for the diagnosis in these conditions.

*Conclusion*: Here we discuss a number of rare congenital HPT axis disorders in which TSH is normal, but FT4 is low, and provide a clinical algorithm to distinguish between these disorders.

**Table:** 

**What is Known:** *• A single thyroid-stimulating hormone (TSH) measurement is an appropriate screening method for primary hypothyroidism.* *• For central hypothyroidism and rare thyroid hormone receptor and transporter defects a free thyroxine (FT4) measurement is essential for the diagnosis because TSH is usually normal.*	
**What is New:** *• Here we present a new problem-oriented clinical algorithm including a diagnostic flow-chart for low FT4 and normal TSH in infants and children.*

**What is Known:**

*• A single thyroid-stimulating hormone (TSH) measurement is an appropriate screening method for primary hypothyroidism.*

*• For central hypothyroidism and rare thyroid hormone receptor and transporter defects a free thyroxine (FT4) measurement is essential for the diagnosis because TSH is usually normal.*

**What is New:**

*• Here we present a new problem-oriented clinical algorithm including a diagnostic flow-chart for low FT4 and normal TSH in infants and children.*

## Introduction

Biochemical evaluation for suspicion of acquired thyroid disease—i.e. hypothyroidism or hyperthyroidism—in adults starts with measurement of serum thyroid-stimulating hormone (TSH). A high and low TSH is diagnostic or at least indicative of these diagnoses. Of course, this interpretation assumes a “healthy” hypothalamus and pituitary gland. Measuring only TSH misses central (i.e. hypothalamic/pituitary) hypothyroidism, characterized by a low free thyroxine (FT4) and an inappropriately low, normal, or slightly elevated TSH (mostly within the normal range). Still, greater cost-effectiveness can be achieved by only measuring TSH to screen for hypothyroidism and hyperthyroidism, since the likelihood of undiagnosed hypothalamic or pituitary conditions in adults without a suggestive medical history is extremely low.

When considering hypothyroidism in infants and children, not only acquired but also congenital hypothalamus-pituitary-thyroid (HPT) axis disorders need to be considered, including primary and central congenital hypothyroidism, and thyroid hormone receptor and transporter defects. Some paediatric patient groups have a higher risk of developing autoimmune thyroid disease, such as patients with a strong family history of autoimmune disease, Down syndrome, Klinefelter syndrome, or another autoimmune disease. The initial screening for thyroid disease in these children may be performed by a single TSH measurement. This also applies to older (post-pubertal) children, as autoimmune thyroiditis is the most likely cause of their thyroid disease. A single TSH measurement should especially suffice in children with clear signs of autoimmune thyroid disease (e.g. goitre, Graves’ ophthalmopathy, or myxedema). In younger children, the likelihood of congenital HPT axis disorders is much higher, while complaints of hypothyroidism can be vague or absent [1]. Thus, a combined TSH and FT4 measurement should be performed in order not to miss these conditions. Signs and symptoms that should always prompt an additional FT4 measurement because of the association with central hypothyroidism are given in Table 1.

**Table 1:** Signs and symptoms (red flags) that should prompt TSH and FT4 measurement

Medical history of brain/pituitary damage (tumour, surgery, irradiation, trauma) Headaches, neurological complaints, visual disturbances Neonatal hypoglycaemia, prolonged neonatal jaundice, breech delivery Micropenis and/or undescended testes Midline defects (solitary central incisor, cleft palate, ocular abnormalities) Developmental problems Growth retardation	

Here we discuss the rare congenital HPT axis disorders in which FT4 is low and TSH is normal and provide a clinical algorithm to distinguish between these conditions.

## Differential diagnosis

The differential diagnosis of a low FT4 accompanied by a normal TSH in infants and children may be divided into transient and permanent conditions (Table 2).

**Table 2:** Differential diagnosis of a low FT4 and normal TSH in infants and children

1. Transient conditions with low FT4 and normal TSH • Non-thyroidal illness syndrome (NTIS) • Transient hypothyroxinaemia of prematurity (THOP) • Medication ○ Anti-epileptic drugs • Transient central hypothyroidism due to maternal Graves’ disease 2. Permanent conditions with low FT4 and normal TSH • Central hypothyroidism (congenital/acquired) • Allan-Herndon-Dudley syndrome/MCT8 deficiency (AHDS) • Resistance to thyroid hormone alpha (RTHα)	

### Transient conditions associated with low FT4 and normal TSH

#### Non-thyroidal illness syndrome

Non-thyroidal illness syndrome (NTIS) is characterized by thyroid function test (TFT) abnormalities during severe illness. The biochemical profile of NTIS consists of a low tri-iodothyronine (T3), low T4 and FT4, elevated reverse T3 (rT3), and a normal TSH. It is postulated that NTIS is an evolutionary adaptive HPT response to acute illness, to limit energy expenditure and catabolism [2]. The underlying pathophysiological mechanisms include alterations in tissue-specific deiodinase activity and decreased hypothalamic thyrotropin-releasing hormone (TRH) expression. In addition, fasting during illness (or in anorexia nervosa) further downregulates TRH release through decreased leptin signalling. Since there is no evidence that thyroid hormone replacement is beneficial for patients with critical illness, treatment is not indicated in NTIS.

#### Transient hypothyroxinaemia of prematurity

The primary cause of a low FT4 and normal TSH in prematurely born infants is transient hypothyroxinaemia of prematurity (THOP). The FT4 concentration in the first weeks after birth is inversely related to gestational age and birth weight. Several factors have been proposed to play a role in THOP, including HPT axis immaturity, loss of maternal thyroid hormone supply, thyroid gland immaturity, abnormal thyroid hormone metabolism, and medication (e.g. dopamine infusion) [3]. Meanwhile, NTIS-related mechanisms also frequently contribute to TFT abnormalities in prematurely born infants, e.g. digestive tract immaturity (undernutrition) and low gestational age-associated morbidity such as respiratory distress syndrome. Retrospective cohort studies evaluating neurodevelopmental outcomes of THOP have found variable negative long-term effects of low thyroid hormone concentrations, while some studies demonstrated that THOP is not associated with a poorer outcome. Although it is undisputed that thyroid hormones are important for brain development, whether thyroid hormone treatment is necessary in THOP remains a subject of debate [3].

Another TFT abnormality observed in preterm infants—and especially those with low birth weights—is a delayed rise of TSH in case of congenital primary hypothyroidism [4]. It was shown that, probably due to HPT axis immaturity, the first TSH elevation occurs after more than 20 days.

Delayed TSH rise, THOP, and NTIS may lead to false negative as well as false positive results in neonatal screening programs for congenital hypothyroidism. In a TSH-based neonatal screening program, primary congenital hypothyroidism may be missed due to a lack of an appropriate TSH elevation, while in thyroxine (T4)-based screening programs, central congenital hypothyroidism may be unjustly suspected (low T4 with normal TSH). Therefore, for premature and low-birth infants and for infants with illness, it is recommended to repeat TSH and FT4 measurements at term-corrected gestational age or at discharge from the hospital as an additional screening for congenital hypothyroidism.

#### Medication

Medication may influence TFTs. Anti-epileptic drugs are known to decrease T4 and FT4. In a cross-sectional study of thyroid function in epilepsy patients, carbamazepine treatment and anti-epileptic drug polytherapy were significant risk factors for low FT4 and normal TSH [5]. There is no association between use of anti-epileptic drugs and complaints or signs of hypothyroidism. Anti-epileptic drugs may cause a low FT4 in combination with a normal TSH by various reversible mechanisms, mainly involving hepatic cytochrome p450 induction (increase in thyroid hormone metabolism). Alterations of the HPT axis and thyroid hormone-binding proteins have also been suggested.

#### Transient central hypothyroidism due to maternal Graves’ disease

Due to the placental transfer of TSH-receptor-stimulating antibodies (TRab), maternal Graves’ disease may lead to foetal and/or neonatal hyperthyroidism. This form of hyperthyroidism affects one to two percent of infants of mothers with past or active Graves’ disease and usually resolves between 1 and 3 months of life, after the clearance of maternal TRab from the neonatal circulation [6]. Infants born to mothers with inadequately treated Graves’ disease may also develop central hypothyroidism, probably due to the prolonged foetal/neonatal exposure to excess thyroid hormones affecting maturation of the HPT axis. In these cases, central hypothyroidism may occur immediately after birth or after a period of neonatal hyperthyroidism. Usually the central hypothyroidism is transient although it may persist in up to 30% of affected children [6].

### Permanent conditions associated with low FT4 and normal TSH

#### Central hypothyroidism

In central hypothyroidism, thyroid hormone production is suboptimal due to insufficient TSH stimulation of an otherwise normal thyroid gland [7]. Acquired causes of central hypothyroidism include damage to the pituitary gland or hypothalamus by invasive/infiltrative lesions (e.g. pituitary tumour, meningioma, Langerhans cell histiocytosis), cranial surgery, cranial irradiation, trauma, and autoimmune inflammation (lymphocytic adenohypophysitis). In general, the medical history will be helpful in diagnosing these acquired conditions. Recognising central congenital hypothyroidism is often complicated due to the lack of such a medical history. Central congenital hypothyroidism may occur in isolation (25% of cases) but is more often accompanied by additional pituitary hormone deficiencies (75%). Most children with multiple pituitary hormone deficiency (MPHD) exhibit a pituitary malformation characterized by an absent or thin pituitary stalk, a hypoplastic anterior pituitary lobe, and an ectopic posterior pituitary lobe, known as pituitary stalk interruption syndrome (PSIS) [1]. Most cases of PSIS occur in isolation although they may be accompanied by additional midline brain abnormalities.

Variants in genes encoding transcription factors involved in pituitary formation and pituitary cell differentiation are found in less than 5% of patients with MPHD (*HESX1, LEPR, LHX3, LHX4, OTX2, POU1F1, PROP1, SOX3*). In contrast, a genetic cause is often found in patients with isolated central hypothyroidism [1]. The five genes associated with isolated congenital central hypothyroidism are thyrotropin-releasing hormone receptor gene (*TRHR*), thyroid-stimulating hormone β-subunit gene (*TSHB*), and the more recently described genes *IGSF1, TBL1X*, and *IRS4* [8, 9].

#### Allan-Herndon-Dudley syndrome (MCT8 deficiency)

Allan-Herndon-Dudley syndrome (AHDS) is an X-linked condition caused by pathogenic variants in the thyroid hormone transporter monocarboxylate transporter 8 (MCT8) gene *SLC16A2* [10]. MCT8 transports thyroid hormone across the blood-brain barrier and into the neurons. Affected males have markedly high serum T3 levels. The clinical picture is the result of a lack of intracerebral thyroid hormone together with peripheral thyrotoxicosis. Patients are born with normal height and weight but rapidly develop severe hypotonia. Due to swallowing difficulties, patients have feeding problems and early onset failure to thrive. Because of axial hypotonia, patients are unable to sit or stand. Later, hypotonia progresses to spastic quadriplegia and dystonia. Peripheral thyrotoxicosis causes tachycardia and contributes to low body weight and muscle wasting. The biochemical profile consists of low (F)T4, normal, or mildly elevated TSH, markedly elevated T3, and reduced rT3. This resembles laboratory findings in patients with thyroid hormone insensitivity due to *THRA* variants (described below) although T3 levels are somewhat higher in MCT8 deficiency. FT4 concentrations in heterozygous females are reportedly low-normal. To date there is no effective treatment for the severe neurological disabilities, although the T3 analogue TRIAC (3,3′,5-tri-iodothyroacetic acid or tiratricol) seems a promising therapeutic agent which is currently being investigated in prospective clinical trials.

#### Resistance to thyroid hormone alpha

Thyroid hormone resistance due to pathogenic variants in the thyroid hormone receptor (TR)-alpha gene (*THRA*) is a very rare condition resulting in low FT4 and normal TSH [11]. Due to reduced thyroid hormone action in tissues expressing TR-alpha, such as the bone, the gastrointestinal tract, heart, and haematological and nervous systems, patients present with growth retardation, developmental delay, and constipation. TFTs are characterized by mildly reduced (F)T4, slightly elevated T3, and low rT3. TSH is within normal ranges because TR-alpha is not involved in HPT feedback. In the few patients described so far, treatment with thyroxine had variable effects, though younger patients have shown improvement of growth and motor development [11].

## Diagnostic work-up of low FT4 and normal TSH in infants and children

**Fig. 1:**
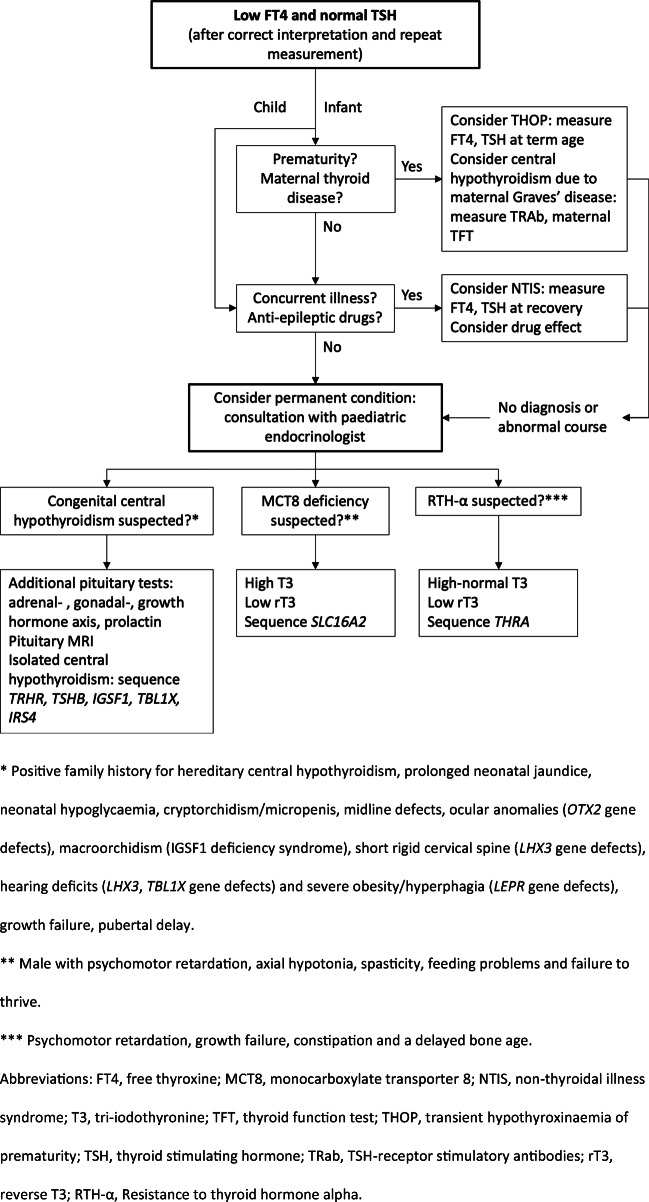
Diagnostic work-up of a low FT4 and normal TSH in infants and children

The diagnostic work-up of low FT4 and normal TSH based on the differential diagnosis is depicted in Fig. 1. When faced with a child with low FT4 and normal TSH, the first step should be to remeasure FT4 and TSH. Especially in the absence of the previously defined red flags indicative of congenital or acquired central hypothyroidism (Table 1), a second FT4 measurement will often yield a normal result. Further, the use of an appropriate assay- and age-specific reference range is important. Caution is advised when interpreting TFTs of very young infants as the reference range for FT4 changes in the first 2–3 months of life [12]. At the age of 2 weeks, the lower limit of the reference range was 15 pmol/L in an FT4 assay with an adult reference range of 12 to 22 pmol/L [12]. Another pitfall of the FT4 reference range is that values around the lower limit of the reference range cannot accurately rule in or rule out mild hypothyroidism. It must be noted that a low FT4 is theoretically present in 2.5% of euthyroid people (the reference range is based on a Gaussian distribution). Likewise, a low-normal FT4 (within the reference range) can still be a sign of a congenital HPT disorder. Immunoassay interference should always be considered in the case of an unexpected thyroid function test result. However, in general, immunoassay interference leads to falsely elevated TSH or to the combination of a falsely reduced TSH and elevated FT4. Still, immunoassays present a negative bias compared to the more accurate equilibrium dialysis-based assays [13]. Performing another FT4 measurement on such a platform may also help in resolving the problem of low FT4 and normal TSH.

In newborn infants, maternal thyroid status should be reviewed as transient central hypothyroidism may be caused by unrecognized or inadequately treated maternal Graves’ disease. Furthermore, the medical history must be interrogated for gestational age, birth weight, use of medication, and a family history of central hypothyroidism. As central hypothyroidism is frequently part of multiple pituitary hormone deficiencies, there should be attention for signs and symptoms of hypopituitarism (Table 1) [1].

In the medical history, an important issue to inquire about is concomitant illness and its severity. If NTIS is suspected, the timing and course of illness are relevant. TFTs are generally not indicated during illness because of NTIS. However, it cannot always be avoided, for example, when routine neonatal screening is scheduled. In case of NTIS, low FT4 is transient, and FT4 and TSH should be remeasured when the child has recuperated. If necessary, for example, in case of prolonged illness, additional T3 and rT3 measurements (decreased and elevated in NTIS, respectively), will aid in confirming NTIS. If FT4 fails to normalize while the child is clinically recuperating, central hypothyroidism needs to be considered.

Since congenital HPT axis disorders are rare, we advise consultation with and possibly referral to a paediatric endocrinologist when performing additional diagnostic tests, including genetic testing. In order to screen for the rare conditions MCT8 deficiency and RTH-α, we advise performing a complete thyroid hormone profile (including T3 and rT3) before initiating levothyroxine treatment. High T3 may indicate MCT8 deficiency or RTH-α.

In summary, when screening for thyroid disease in children, in addition to TSH measurement, an FT4 should be considered in order not to miss rare congenital forms of hypothyroidism (Table 1). The differential diagnosis of low FT4 and TSH in children includes transient and permanent conditions (Table 2). A repeat FT4 and TSH measurement (if necessary with a different assay) and correct interpretation of the FT4 concentration are the first steps of the diagnostic work-up. Next, a detailed medical history, physical examination, and occasionally additional thyroid hormone measurements (T3 and rT3) are necessary (Fig. 1). Due to the very rare nature of the various congenital HPT axis disorders, consultation with a paediatric endocrinologist is advised to come to a final diagnosis.

## Data Availability

N/A.

## Abbreviations

FT4: Free thyroxine
HPT: Hypothalamic-pituitary-thyroid
MCT8: Monocarboxylate transporter 8
MPHD: Multiple pituitary hormone deficiencies
NTIS: Non-thyroidal illness syndrome
PSIS: Pituitary stalk interruption syndrome
T3: Tri-iodothyronine
TBG: Thyroxine-binding-globulin
TFT: Thyroid function test
THOP: Transient hypothyroxinaemia of prematurity
TSH: Thyroid-stimulating hormone
TRab: TSH-receptor stimulatory antibodies
TRH: Thyrotropin-releasing hormone
rT3: Reverse T3
RTH-α: Resistance to thyroid hormone alpha

## Code availability

N/A.
